# Constitutive phosphorylation of the FOXO1 transcription factor in gastric cancer cells correlates with microvessel area and the expressions of angiogenesis-related molecules

**DOI:** 10.1186/1471-2407-11-264

**Published:** 2011-06-22

**Authors:** Sue Youn Kim, Jiyeon Yoon, Young San Ko, Mee Soo Chang, Jong-Wan Park, Hee Eun Lee, Min A Kim, Ji Hun Kim, Woo Ho Kim, Byung Lan Lee

**Affiliations:** 1 Department of Anatomy, Seoul National University College of Medicine, 103 Daehak-ro, Jongno-gu, Seoul 110-799, Korea; 2 Department of Pathology, Seoul National University College of Medicine, 103 Daehak-ro, Jongno-gu, Seoul 110-799, Korea; 3 Department of Pharmacology, Seoul National University College of Medicine, 103 Daehak-ro, Jongno-gu, Seoul 110-799, Korea; 4 Ischemic/Hypoxic Disease Institute Medical Research Center, Seoul National University College of Medicine, 103 Daehak-ro, Jongno-gu, Seoul 110-799, Korea; 5 Department of Pathology, Asan Medical Center, Ulsan University College of Medicine, 388-1 Pungnap-2-dong, Songpa-gu, Seoul 138-736, Korea; 6 Cancer Research Institute, Seoul National University College of Medicine, 103 Daehak-ro, Jongno-gu, Seoul 110-799, Korea

**Keywords:** pFOXO1, angiogenesis, gastric cancer, immunohistochemistry, tissue array analysis

## Abstract

**Background:**

Although FOXO transcription factors may have an anti-angiogenic role, little is known about their role in tumor angiogenesis. The present study was performed to investigate the correlation between the constitutive expression of phosphorylated FOXO1 (pFOXO1) and angiogenesis in gastric cancer.

**Methods:**

Immunohistochemistry was performed on tissue array slides containing 272 gastric carcinoma specimens, and the correlations between the cytoplasmic pFOXO1 expression in gastric cancer cells and CD34-immunopositive microvessel area (MVA) or the expressions of angiogenesis-related molecules were analyzed. *In vitro *analyses with Western blotting and semiquantitative reverse transcription-polymerase chain reaction were performed using the stable SNU-638 gastric cancer cell line transfected with lentivirus-delivered FOXO1 short hairpin RNA.

**Results:**

The cytoplasmic expression of pFOXO1 in tumor cells was observed in 85% of gastric carcinoma cases, and was found to be positively associated with higher MVA (*P *= 0.048). Moreover, pFOXO1 expression was positively correlated with the expressions of several angiogenesis-related proteins, including hypoxia inducible factor-1α (HIF-1α, *P *= 0.003), vessel endothelial growth factor (*P *= 0.004), phosphorylated protein kinase B (*P *< 0.001), and nuclear factor-κB (*P *= 0.040). In contrast, the expression of pFOXO1 was not correlated with that of phosphorylated signal transducer and activator of transcription 3 or β-catenin. In addition, cell culture experiments showed that FOXO1 suppression increased the mRNA and protein expressions of HIF-1α.

**Conclusion:**

Our results suggest that pFOXO1 expression in cancer cells plays a role in gastric cancer angiogenesis via mechanisms involving various angiogenesis-related molecules. Animal experiments are needed to confirm the anti-angiogenic role of FOXO1 in human gastric cancer.

## Background

The FOXO (Forkhead box, class O) is a subfamily of forkhead transcription factor and consists of FOXO1A (FKHR: Forkhead in rhabdomyosarcoma, also known as FOXO1), FOXO3A (FKHR-like 1), MLLT7 (AFX: acute-lymphocytic-leukaemia-1 fused gene from chromosome X, also known as FOXO4) and FOXO6 [1]. Phosphorylated FOXOs could not exhibit transcriptional activity because the phosphorylated forms are exported from the nucleus [2]. FOXOs are now emerging as an important family of proteins that are implicated in the regulations of several biological processes, including the stress resistance, metabolism, cell cycle, apoptosis, and DNA repair [3]. Thus, dysregulations of these proteins may ultimately lead to disease such as cancer [4].

Inactivation of FOXOs has been reported in various cancers, including breast cancer, prostate cancer, chronic myelogenous leukemia, glioblastoma, rhabdomyosarcoma, and leukemia [5]. Previous studies have shown that the anti-tumor activity of FOXOs comes from their pro-apoptotic [6-8] and inhibitory cell cycle effects [3,9]. Recently, abnormal vascular development was observed in embryonic FOXO1-deficient mice [10], and in another study, it was found that FOXO1 and FOXO3A are critical regulators of endothelial sprout formation and migration *in vitro *[11]. Accordingly, it was suggested that the inactivation of FOXOs might regulate angiogenesis [12]. However, the role of FOXOs in tumor angiogenesis has not been investigated.

Gastric cancer is one of the most common cancers, and the major cause of cancer-related death worldwide [13]. However, the molecular mechanism underlying gastric tumor angiogenesis remains unclear. Previously, it was reported that the phosphorylated inactive form of FOXO1 (pFOXO1) was constitutively expressed in gastric cancer and that this was clinically significant [14]. To investigate the correlation between pFOXO1 and angiogenesis in gastric cancer, the present study performed immunohistochemical tissue array analysis using 272 surgically excised human gastric cancer specimens. Subsequently, we analyzed the correlation between the expression of pFOXO1 and microvessel area (MVA) or the expressions of several angiogenesis-related molecules, including hypoxia inducible factor-1α (HIF-1α), vessel endothelial growth factor (VEGF), phosphorylated protein kinase B (pAKT), and nuclear factor-κB (NF-κB), phosphorylated signal transducer and activator of transcription 3 (pSTAT3) and β-catenin. In addition, we performed cell culture experiments after establishing a stable gastric cancer cell line transfected with lentivirus-delivered FOXO1 short hairpin RNA (shRNA).

## Methods

### Patients and Samples

The files of 272 surgically resected gastric cancer cases examined at the Department of Pathology, Seoul National University College of Medicine from 1 January to 30 June 1995 were analyzed. Age, sex, tumor location and pTNM stage were evaluated by reviewing the medical records and pathological reports [15]. The mean age of the patients was 54.8 years, and 93.3% of the patients had undergone curative resection. The cases enrolled in this study included 193 advanced and 79 early gastric carcinomas. According to the UICC criteria, there were 112 cases in stage I, 53 cases in stage II, 63 cases in stage III, and 44 cases in stage IV. No patient had received preoperative chemotherapy or radiotherapy. Glass slides were reviewed to determine histological type according to the WHO and Lauren's classification. his series included 102 intestinal types, 166 diffuse types, and 4 mixed types. Clinical outcomes were followed from the date of surgery to either the date of death or December 1^st^, 2000, resulting in the follow-up period ranged from 1 month to 72 months (mean, 51 months). Cases lost to follow up and those resulting in death from any cause other than gastric cancer were censored for survival rate analysis. This protocol was reviewed and approved by the Institutional Review Board of Seoul National University (Approval No. C-0511-519-163).

### Tissue array methods

Six array blocks obtained from patients with a gastric cancer were prepared as described previously (Superbiochips Laboratories, Seoul, Korea) [16]. Briefly, core tissue biopsies (2 mm in diameter) were taken from individual paraffin-embedded gastric tumors (donor blocks) and arranged in the new recipient paraffin blocks (tissue array block) using a trephine apparatus. As we have reported previously [16], the staining results of the different intratumoral areas of gastric carcinomas in these tissue array blocks showed an excellent agreement. A core was chosen from each case for analysis. We defined an adequate case as a tumor occupying more than 10% of the core area. Sections of 4 μm thickness were cut from each tissue array block, deparaffinized, and dehydrated.

### Immunohistochemistry

Immunohistochemical staining was performed as described previously using a streptavidin peroxidase procedure (avidin-biotin complex method) after antigen retrieval using an autoclave [17]. Anti-phospho-Ser256-FOXO1 (1:50) from Cell Signaling Technology (Beverly, MA, USA) was used as the primary antibody. Other primary antibodies used were anti-CD34 (1:300, Immunotech, Cedex, France), anti-HIF-1α (donated by JW Park at SNU, Seoul, Korea), anti-VEGF (1:250, Santa Cruz Biotechnology, Santa Cruz, CA, USA), anti-phospho-Ser473-AKT (1:100, New England Biolabs, Beverly, MA, USA), anti-NF-κB p65 (1:50, Santa Cruz Biotechnology), anti-phospho-Tyr705-STAT3 (1:50, Cell Signaling Technology) and anti-β-catenin (1:200, Transduction, Lexington, KY, USA). The results of immunostaining were evaluated by two pathologists (JHK and MSC), who were blinded to the origin of the samples. The concordance rates were generally high ranging from 75.7% (κ = 0.328, *P *< 0.001) to 87.5% (κ = 0.591, *P *< 0.001). Discrepancies were resolved by reaching consensus. For statistical analysis, the results of immunostaining for proteins were considered positive if immunoreactivity was seen in ≥ 10% (cytoplasmic pFOXO1, cytoplasmic VEGF, cytoplasmic and nuclear pAKT, nuclear NF-κB and nuclear β-catenin), ≥ 5% (nuclear HIF-1α) or ≥ 1% (nuclear pSTAT3) of the neoplastic cells.

### Evaluation of microvessel area (MVA)

Vessel areas immunostained with an anti-CD34 antibody in gastric cancer specimens were determined as described previously [17]. Tissue sections were examined by two independent observers (SYK and JY). Briefly, the three most highly vascularized areas in areas of tumor sections were selected under × 100 magnification and photographs of CD34-immunopositive microvessel vessels in tumor sections were taken under × 200 magnification using light microscopy. The cross-sectional areas of CD34-immunopositive structures (i.e. MVA) were quantified by capturing images, converting them to gray scale and analyzing CD34-stained areas using NIH Image Analysis software (version 1.62; National Institute of Health, Bethesda, MD, USA) after setting one consistent intensity threshold for all slides. Then, CD34-positive areas were expressed as pixels squared per high-power field.

### Cell culture

A human gastric cancer cell line SNU-638 was obtained from the Korean Cell Line Bank (Seoul, Korea), cultured in RPMI-1640 medium (Life Technologies, Grand Island, NY, USA) containing 10% fetal bovine serum (Life Technologies), and maintained in a 37°C humidified incubator containing 95% air and 5% CO_2_. To examine the effect of FOXO1 suppression on HIF-1α expression, cells were incubated under either normoxic or hypoxic conditions.

### Lentivirus-mediated shRNA silencing of FOXO1

FOXO1 shRNA lentiviral particles and non-targeting shRNA control particles were purchased (Sigma, St Louis, MO, USA). The sequence of the shRNA targeting FOXO1 used in the present study is the following: CCGGGCCTGTTATCAATCTGCTAAACTCGAGTTTAGCAGATTGATAACAGGCTTTTTG. The non-targeting shRNA control particles contain 4 basepair mismatches within the short hairpin sequence to any known human or mouse gene. The viral infection was performed by incubating SNU-638 gastric cancer cells in the culture medium containing lentiviral particles for 12 h in the presence of 5 μg/ml Polybrene (Santa Cruz Biotechnology). Pooled puromycin (2 μg/ml)-resistant cells were harvested and stored for further analysis.

### Western blotting

Cell lysates were prepared in 100-200 μl of 1 × SDS lysis buffer [125 mM Tris-HCl (pH 6.8), 4% SDS, 0.004% bromophenol blue, and 20% glycerol] and then boiled for 10 min. Protein contents were measured using BCA Protein Assay Reagent (Pierce, Rockford, IL, USA). Samples were diluted with 1 × lysis buffer containing 1.28 M β-mercaptoethanol, and equal amounts of protein were loaded onto 8-12% SDS-polyacrylamide gels. Proteins were electrophoretically transferred to PVDF or nitrocellulose membranes, and membranes were then blocked with 5% nonfat dry milk in PBS/Tween-20 (0.1%, vol/vol) at 4°C overnight. They were then incubated with a primary antibody against FOXO1 (1:1000; Cell Signaling Technology), HIF-1α (1:1000; donated by JW Park), or β-actin (1:1000; Sigma) for 2 h. They were then incubated with a corresponding secondary antibody, either horseradish peroxidase-conjugated anti-rabbit IgG (1:2000; Zymed, San Francisco, CA, USA) or anti-mouse IgG (1:2500; Santa Cruz Biotechnology) for 2 h, and enhanced chemiluminescence (ECL) (Amersham Biosciences, Arlington Heights, IL, USA) was used for the visualization of the immunoreactive proteins. Equal loading of the protein was confirmed by β-actin.

### Semiquantitative reverse transcription-polymerase chain reaction (SQ RT-PCR)

To quantify mRNA levels, we used a highly sensitive, SQ RT-PCR method. Total RNAs were isolated using TRIZOL reagent purchased from Invitrogen (Carlsbad, CA, USA), and 1 μg of RNAs were reverse-transcribed at 48°C for 30 min. Complementary DNAs were amplified over 18 PCR cycles (94°C for 30 sec, 52°C for 30 sec, and 70°C for 30 sec) in a reaction mixture containing 5 mCi (α-^32^P)dCTP (NEN, Boston, MA, USA). The resulting PCR fragments (5 ml) were electrophoresed on a 2% agarose gel at 100 V in 1 × TAE, and the gels were dried and autoradiographed. Primer sequences were 5'-CCCCAGATTCAGGATCAGACA-3' and 5'-CCATCATGTTCCATTTTTCGC-3' for HIF-1α, and 5'-ACACCTTCTACAATGAGCTG-3' and 5'-CATGATGGAGTTGAAGGTAG-3' for β-actin.

### Statistical analysis

All statistical analyses were conducted using SPSS version 11.0 software (SPSS, Chicago, IL, USA). The significance of correlation between the expressions of pFOXO1 and other proteins was determined by the chi-squared test. MVAs were expressed as means ± S.D. and the relationship between pFOXO1 expression and MVA was analyzed using the two-tailed Student's *t*-test. Survival curves were estimated using the Kaplan-Meier product-limit method, and the significance of differences between the survival curves was determined using the log-rank test. Regarding the survival curve, the MVA score was considered positive when the value was higher than the mean value and vice versa. *P *< 0.05 was considered significant.

## Results

### Expressions of pFOXO1 and angiogenesis-related proteins in gastric cancer specimens

**Figure 1:**
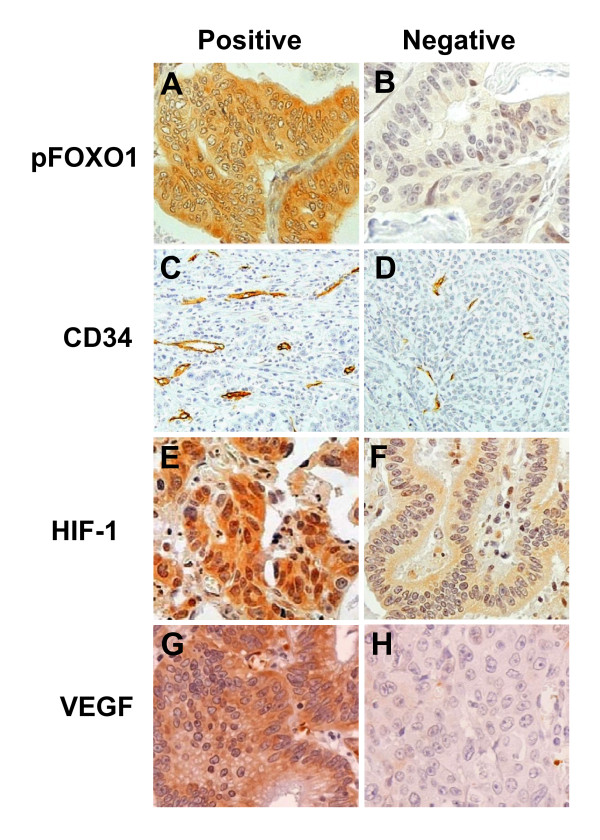
**Representative immunohistochemical findings in gastric cancer tissues**. **A: **gastric carcinoma cells showing cytoplasmic pFOXO1A expression with or without nuclear staining (× 400). **B: **gastric carcinoma cells without cytoplasmic pFOXO1A expression (× 400). **C: **a representative gastric tumor section showing high MVA (× 200). **D: **a representative gastric tumor section showing low MVA (× 200). **E: **gastric carcinoma cells showing nuclear HIF-1α expression with or without cytoplasmic staining (× 400). **F: **gastric carcinoma cells without nuclear HIF-1α expression (× 400). **G: **gastric carcinoma cells showing cytoplasmic VEGF expression (× 400). **F: **gastric carcinoma cells without cytoplasmic VEGF expression (× 400).

Immunohistochemical staining was used to investigate the correlation between pFOXO1 expression and angiogenesis in gastric cancer. Figure 1 shows representative immunohistochemical features of proteins in gastric cancer tissues. pFOXO1 (Figure 1A and 1B) and HIF-1α (Figure 1E and 1F) were found to be expressed in both the nuclei and cytoplasm of tumor cells. Regarding pFOXO1 staining, cells showing distinct cytoplasmic staining, regardless of the presence of nuclear staining, were considered to express pFOXO1 constitutively, whereas those with nuclear HIF-1α expression, regardless of the presence of cytoplasmic staining, were considered to exhibit activated HIF-1α. Since immunostaining was performed with an antibody against an inactivated form of FOXO1 (pFOXO1), an increase in pFOXO1 expression was interpreted as a decrease in FOXO1 activation. Consequently, correlations between pFOXO1 and other proteins are opposite to those between activated FOXO1 and these proteins. Constitutive pFOXO1 expression was observed in 230 (85%) of the 272 gastric cancer cases examined.

### Association between the expression of pFOXO1 and MVA

MVA is a widely accepted measure of neoangiogenetic activity in cancer. Mean CD34-positive microvessel areas were found to be significantly larger in pFOXO1-positive tumors (Figure 1C) than in pFOXO1-negative tumors (Figure 1D) (*P *= 0.048) (Table 1).

**Table 1:** Correlation between cytoplasmic pFOXO1 expression in tumor cells and MVA in gastric cancer

		MVA		
		
	Cases	**Mean ± S.D**.	**S.E**.	*P*
pFOXO1				
Positive	212	10.77 ± 8.8	0.60347	0.048*
Negative	39	7.87 ± 5.3	0.84711	

Abbreviations: MVA, microvessel area; pFOXO1, phosphorylated Forkhead in rhabdomyosarcoma.

* Considered to be statistically significant.

### pFOXO1 expression and MVA in relation to prognosis

**Figure 2:**
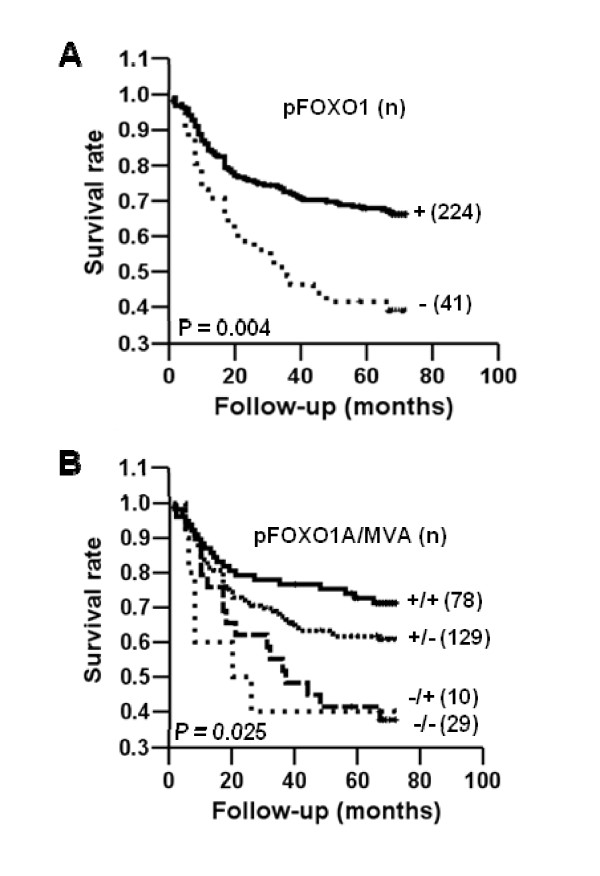
**Kaplan-Meier curves for gastric cancer patient survival according to immunohistochemical status**. **A: **Patients with a pFOXO1-positive carcinoma (solid line) showed a more favorable prognosis than those with a pFOXO1-negative carcinoma (dotted line) (*P *= 0.004). **B: **Patients with a pFOXO1-positive carcinoma and with higher MVA (solid line) showed the best outcome (*P *= 0.025).

Our analysis of the 272 gastric cancer patients revealed that those with pFOXO1 expression had a significantly higher survival rate than those without pFOXO1 expression (*P *= 0.004) (Figure 2A). In our analysis of the combined status of the expression of pFOXO1 and MVA in relations to prognosis, patients with a pFOXO1-positive tumor and higher MVA had better outcome than patients with the other combinations (*P *= 0.025) (Figure 2B).

### Correlation between the expression of pFOXO1 and the expression of angiogenic proteins in surgical gastric cancer samples

To investigate the positive correlation found between pFOXO1 expression and angiogenesis further, we analyzed the associations between the expression of pFOXO1 and the expressions of other potential mediators of angiogenesis. Table 2 shows that pFOXO1 expression was positively correlated with the expressions of the critical angiogenic regulators HIF-1α (*P *= 0.003) and VEGF (*P *= 0.004). Furthermore, pFOXO1 expression was significantly and positively correlated with the expressions of other pro-angiogenic molecules, that is, pAKT (*P *< 0.001) and NF-κB (*P *< 0.040), but not with those of pSTAT3 and β-catenin.

**Table 2:** Correlation between the expression of pFOXO1 and that of angiogenesis-related proteins in tumor cells in gastric cancer

	pFOXO1		
		
	Positive (%)	Negative (%)	*P*
pFOXO1			
HIF-1α			
Positive	64 (96)	3 (4)	0.003*
Negative	133 (80)	34 (20)	
VEGF			
Positive	87 (93)	7 (7)	0.004*
Negative	110 (79)	30 (21)	
pAKT			
Positive	169 (90)	18 (10)	< 0.001*
Negative	28 (60)	19 (40)	
NF-κB			
Positive	38 (95)	2 (5)	0.040*
Negative	159 (82)	35 (18)	
pSTAT3			
Positive	49 (89)	6 (11)	0.255
Negative	148 (83)	31 (17)	
β-catenin			
Positive	38 (86)	6 (14)	0.661
Negative	159 (84)	31 (16)	

Abbreviations: pFOXO1, phosphorylated Forkhead in rhabdomyosarcoma (cytoplasmic staining);

HIF-1α, hypoxia inducible factor-1α (nuclear staining); VEGF, vessel endothelial growth factor (cytoplasmic staining); pAKT, phosphorylated protein kinase B (cytoplasmic and nuclear staining);

NF-κB, nuclear factor-κB (nuclear staining); pSTAT3, phosphorylated signal transducer and activator of transcription 3 (nuclear staining); β-catenin (nuclear staining)

* Considered to be statistically significant.

### Correlations between pFOXO1 expression in tumor cells and MVA in HIF-1α-positive and HIF-1α-negative tumors and in VEGF-positive and VEGF-negative tumors

To remove the possible confounding effects of HIF-1α and VEGF on the relationship between cytoplasmic pFOXO1 expression and angiogenesis in gastric cancer, we divided samples into two groups according to the expression of HIF-1α or VEGF (Table 3). Although pFOXO1 expression positively correlated with MVA in overall patients (*P *= 0.048), there was no significant correlation after we removed the effect of HIF-1α or VEGF expression.

**Table 3:** Correlations between the MVA and pFOXO1 expression in tumor cells in HIF-1α-positive and HIF-1α-negative tumors and in VEGF-positive and VEGF-negative tumors

	MVA		
		
	Cases	**Mean ± S.D**.	*P*
**HIF-1 positive**			
pFOXO1			
Positive	61	12.06 ± 10.9	0.336
Negative	3	5.85 ± 9.0	
**HIF-1 negative**			
pFOXO1			
Positive	130	9.40 ± 7.2	0.332
Negative	33	8.11 ± 5.2	

**VEGF positive**			
pFOXO1			
Positive	84	10.49 ± 8.2	0.667
Negative	6	9.02 ± 4.3	
**VEGF negative**			
pFOXO1			
Positive	107	10.79 ± 9.3	0.071
Negative	30	7.56 ± 5.5	

Abbreviations: pFOXO1, phosphorylated Forkhead in rhabdomyosarcoma (cytoplasmic staining); HIF-1α, hypoxia inducible factor-1α (nuclear staining); VEGF, vessel endothelial growth factor (cytoplasmic staining)

### Effect of FOXO1 inhibition on the expression of HIF-1α in gastric cancer cells in vitro

**Figure 3:**
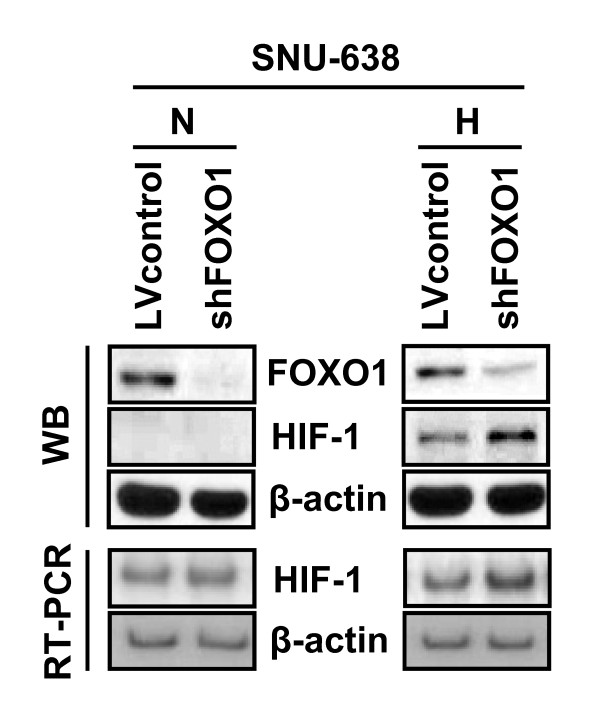
**Effect of FOXO1 inhibition on HIF-1α expression under hypoxic conditions *in vitro***. SNU-638 gastric cancer cells were infected with a lentivirus containing a construct, encoding either FOXO1 shRNA (denoted as shFOXO1) or non-targeting shRNA (denoted as LVcontrol). Expression levels of HIF-1α and β-actin proteins were determined by Western blotting (WB) and the mRNA expressions of HIF-1α and β-actin were determined by SQ RT-PCR (RT-PCR) after cells were exposed to either normoxia (N) or hypoxia (H) for 8 h.

HIF-1α has been reported to increase gastric tumor growth and angiogenesis [18]. Thus, we performed cell culture experiments to further examine the direct relationship between the expressions of FOXO1 and HIF-1α. FOXO1 expression was suppressed by transfecting FOXO1 shRNA into SNU-638 gastric cancer cells, and subsequently, cells were cultured under normoxic or hypoxic conditions. Western blot analysis showed that decreased FOXO1 expression increased HIF-1α protein expression under hypoxic conditions (Figure 3, upper), and RT-PCR showed that decreased FOXO1 expression increased HIF-1α mRNA expression under both normoxic and hypoxic conditions (Figure 3, lower). These results suggest that FOXO1 inhibits HIF-1α expression at the transcriptional level.

## Discussion

Angiogenesis is a complex, multistep process, which is tightly controlled by a balance between pro- and anti-angiogenic factors [19,20]. Although it has been previously reported that FOXO1 in endothelial cells has an anti-angiogenic function [10], the correlation between FOXO1 and tumor angiogenesis remains unknown. In the present study, our results of immunohistochemical tissue array analysis and *in vitro *cell culture experiments suggest that FOXO1 in gastric cancer cells is implicated in gastric tumor angiogenesis. This is the first report, to the best of our knowledge, to show a link between FOXO1 activation in cancer cells and tumor angiogenesis.

Tumor angiogenesis can be investigated in several ways: (i) by measuring microvessel densities (MVDs) or microvessel areas (MVAs) in tumor tissues, (ii) by quantifying angiogenic molecules or angiogenic receptors within tumor tissues, or (iii) by measuring angiogenic factor levels in serum and urine of cancer patients [21-25]. In the present study, we evaluated MVAs (CD34-immunopositive microvessel areas) in gastric tumor sections and the expressions of several angiogenic molecules to investigate angiogenesis.

It is known that vessel area is a critical determinant of blood flow (Poiseuille's law) [26]. Thus, Stoeltzing et al. (2004) measured MVA in tissue sections of gastric cancer xenografts in nude mice to analyze the functional vascular network in gastric cancer [26]. Moreover, in renal cell carcinoma, MVA has been reported to be a more important prognostic marker than MVD [27]. Thus, in present study, MVA was used to investigate the association between pFOXO1 expression and angiogenesis. We found that cytoplasmic pFOXO1 expression in gastric cancer cells was positively correlated with MVA (*P *= 0.048), suggesting that FOXO1 is involved in gastric tumor angiogenesis.

In several previous studies, MVD has been used to assess angiogenesis in human gastric cancer specimens [28-33]. Some of them showed a positive association between high MVD and reduced survival of gastric cancer patients [28-30]. However, Cabuk et al. (2007) reported that MVD was significantly higher in early-stage gastric tumors [33]. Thus, the prognostic significance of MVD in gastric cancer has been inconsistent. In the present study, MVA was found to be significantly higher in early-stage gastric tumors (P < 0.001, data not shown), which agrees with the results of Cabuk et al. (2007). Thus, we speculate that the discrepancies between the results of previous studies [28-30] and the present study do not necessarily stem from the different methods used to measure angiogenesis.

A large number of molecules that either activate or inhibit angiogenesis have been identified. Of these, HIF-1α and VEGF are recognized to be representative marker proteins of angiogenesis, and are known to be associated with pro-angiogenic phenotypes of numerous tumors, including gastric carcinoma [26]. Although, in a previous study, we demonstrated that both pFOXO1 and HIF-1α were constitutively over-expressed in gastric cancer [14,17], the correlation between these transcription factors has not been reported in human cancer. In the present study, large scale immunohistochemical tissue array analysis was performed, and the cytoplasmic pFOXO1 expression in tumor cells was found to be positively correlated with the nuclear expression of HIF-1α (*P *= 0.003) and with the cytoplasmic expression of VEGF (*P *= 0.004) in gastric cancer specimens. However, no significant correlation was found between pFOXO1 expression and MVA after adjusting for the confounding effects of HIF-1α and VEGF. Thus, our results suggest that the positive relationship between pFOXO1 and MVA is dependent on a positive correlation between pFOXO1 and HIF-1α or VEGF.

Recent advances in biology demonstrated that RNAi is a powerful tool in tumor biology investigations. In the present study, FOXO1 expression was inhibited by infecting gastric cancer cells (SNU-638) with a shRNA-expressing lentivirus. In addition, cell culture experiments under hypoxic conditions showed that FOXO1 suppression increased the expressions of HIF-1α at the mRNA and protein levels. These findings suggest that FOXO1 is a signaling molecule upstream of HIF-1α in the angiogenic pathway in gastric cancer, and that gastric tumor angiogenesis could be inhibited, at least in part, by FOXO1 activation.

FOXO1 is a major substrate of AKT. Indeed, our data showed that pFOXO1 expression was closely and positively correlated with the expression of the active form of AKT (pAKT, *P *< 0.001). Since we found previously that AKT activation induced angiogenesis in gastric cancer xenografts [17], our results suggest that AKT/FOXO1 pathway is involved in angiogenesis in gastric cancer. Furthermore, we found that pFOXO1 expression was positively correlated with the nuclear expression of NF-κB (*P *= 0.040), which is also known to induce tumor angiogenesis [34]. However, no association was found between pFOXO1 expression and the expressions of pSTAT3 and nuclear β-catenin in the present study, although they have been reported to be involved in angiogenesis in several cancer types [35,36].

## Conclusion

Our results suggest that pFOXO1 in combination with several pro-angiogenic molecules, could induce the angiogenic phenotypes of gastric cancer. Further *in vivo *animal experiments are needed to confirm these relationships.

## List of abbreviations used

FOXO1: Forkhead in rhabdomyosarcoma; MVA: microvessel area; HIF-1α: hypoxia inducible factor-1α; VEGF: vessel endothelial growth factor; pAKT: phosphorylated protein kinase B; NF-κB: nuclear factor-κB; pSTAT3: phosphorylated signal transducer and activator of transcription; shRNA: short hairpin RNA; SQ RT-PCR: Semiquantitative reverse transcription-polymerase chain reaction

## Competing interests

The authors declare that they have no competing interests.

## Authors' contributions

BLL carried out the design of the study and helped to write, organize and correct the paper. WHK and MAK carried out the patients follow up, SYK and JY carried out the immunohistochemcal staining and drafted the manuscript. JHK and MSC interpreted the immunohistochemical results and performed statistical analysis. YSK performed *in vitro *cell culture experiments. JWP and HEL participated in immunohistochemical staining and the statistical analysis. All authors read and approved the final manuscript.

## Pre-publication history

The pre-publication history for this paper can be accessed here:

http://www.biomedcentral.com/1471-2407/11/264/prepub
